# Promotion of Ni^2+^ Removal by Masking Toxicity to Sulfate-Reducing Bacteria: Addition of Citrate

**DOI:** 10.3390/ijms16047932

**Published:** 2015-04-09

**Authors:** Junwei Qian, Xiaoyu Zhu, Yong Tao, Yan Zhou, Xiaohong He, Daping Li

**Affiliations:** 1 Key Laboratory of Environmental and Applied Microbiology, Chengdu Institute of Biology, Chinese Academy of Sciences & Environmental Microbiology Key Laboratory of Sichuan Province, Chengdu 610041, China; E-Mails: jwqian209@outlook.com (J.Q.); zhuxy@cib.ac.cn (X.Z.); hexh@cib.ac.cn (X.H.); 2 College of Life Science, Sichuan University, Chengdu 610064, China; E-Mail: zhyn807@163.com

**Keywords:** Ni removal, Ni-citrate complex, toxicity masking, lactate, sulfate reduction

## Abstract

The sulfate-reducing bioprocess is a promising technology for the treatment of heavy metal-containing wastewater. This work was conducted to investigate the possibility of promoting heavy metal removal by the addition of citrate to mask Ni^2+^ toxicity to sulfate-reducing bacteria (SRB) in batch reactors. SRB growth was completely inhibited in Ni^2+^-containing medium (1 mM) when lactate served as the sole carbon resource, leading to no sulfate reduction and Ni^2+^ removal. However, after the addition of citrate, SRB grew well, and sulfate was quickly reduced to sulfide. Simultaneously, the Ni-citrate complex was biodegraded to Ni^2+^ and acetate. The NiS precipitate was then formed, and Ni^2+^ was completely removed from the solution. It was suggested that the addition of citrate greatly alleviates Ni^2+^ toxicity to SRB and improves the removal of Ni^2+^, which was confirmed by quantitative real-time PCR targeting dissimilatory sulfite reductase (dsrAB) genes. Analysis of the carbon metabolism indicated that lactate instead of acetate served as the electron donor for sulfate reduction. This study offers a potential approach to increase the removal of heavy metals from wastewater in the single stage SRB-based bioprocess.

## 1. Introduction

Heavy metals, often associated with mine waters and industrial wastewater contributions, have been reported as a cause of many human diseases, such as kidney or nervous system damages and cancers. Sulfate-reducing bioprocess for treating wastewaters from mining and mineral processing are becoming an alternative to conventional chemical treatment [1,2,3]. This anaerobic process is carried out by sulfate-reducing bacteria (SRB) that utilize sulfate as the final electron acceptor for the oxidation of organic compounds (electron donors), resulting in the production of hydrogen sulfide (H_2_S). The removal of metals by SRB is mainly due to the production of highly insoluble precipitates with the biologically produced H_2_S, as shown in Equations (1) and (2) [4]:

(1) $$2{\text{ CH}}_{2}\text{O}+{\text{ SO}}_{4}^{2-}\;\to {\text{ H}}_{2}\text{S}+2{\text{ HCO}}_{3}^{-}\;$$

where CH_2_O = Electron donor;

(2) $${\text{H}}_{2}\text{S}+{\text{M}}^{2+}\;\to \text{MS }\left(\text{s}\right)+\;2{\text{ H}}^{+}\;$$

where M^2+^ = Metal, such as Ni^2+^.

In comparison with traditional chemical treatment, the sulfate-reducing bioprocess could combine the removal of soluble metals and sulfate with low sludge production and cost effectiveness [5].

In recent years, the SRB-based bioprocess has been successfully applied by using single or separated unit processes to treat metal-containing wastewaters [4,5,6]. In the single-stage process, biological sulfide production and chemical metal sulfide precipitation occur simultaneously in the same reactor, whereas in the two-stage configuration, these steps take place separately in different reactors [7]. In comparison with the separated unit process, the single-stage process has the advantage of low capital investment and operational costs [5]. However, the single-stage process may not be viable if the wastewater contains relatively high concentration of heavy metals. In anaerobic semi-continuous stirred tank reactors, when the heavy metal concentration is above the tolerable concentration of SRB, the growth and sulfate-reducing activity of SRB were completely inhibited, and no metal was removed from the solutions [8].

Citric acid is a naturally occurring hydroxycarboxylic acid. Under anaerobic condition, citrate was fermented to formate and acetate, and the produced acetate served as the electron donor for sulfate reduction [9,10]. Citrate is also a multidentate chelating agent, which forms stable metal-citrate complexes with heavy metals [11]. It was reported that citrate effectively masked Ni^2+^ toxicity to *Pseudomonas fluorescens* and *P. alcaliphila* via the formation of non-toxic Ni-citrate complex, and bidentate Ni-citrate complex was biodegraded into Ni^2+^ and CO_2_ under aerobic conditions [12,13,14]. To date, research relating to masking Ni toxicity to SRB via citrate complexation in a single-stage SRB-based process has never been reported.

The aims of this work are to investigate: (i) Whether the addition of citrate can alleviate Ni^2+^ toxicity to SRB; (ii) The promotion of Ni^2+^ removal by masking toxicity to SRB; and (iii) The mechanism of Ni^2+^ masking and removal.

## 2. Results

### 2.1. The Effect of the Addition of Citrate on Ni^2+^ Toxicity

**Figure 1:**
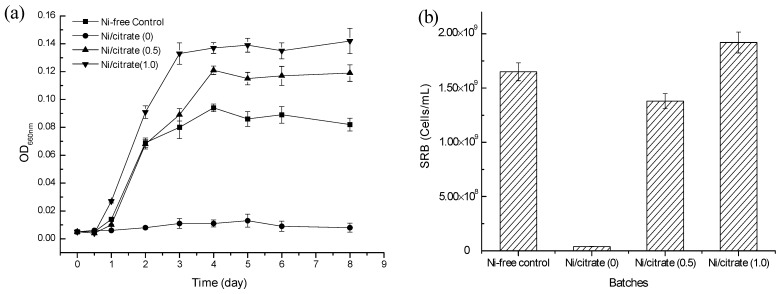
Variations of OD_660_ (**a**) and final sulfate-reducing bacteria (SRB) (**b**) in Ni-free control, Ni/citrate (0), Ni/citrate (0.5) and Ni/citrate (1.0) media (error bars represent one standard deviation of the mean; *n* = 3).

Table 1 shows Ni speciation present in initial Ni/citrate (0), Ni/citrate (0.5) and Ni/citrate (1.0) media after the addition of SRB culture. In the absence of citrate, Ni was present predominantly in the forms of the Ni-lactate complex (43.21%) and free Ni^2+^ (26.20%). In the Ni/citrate (0.5) and Ni/citrate (1.0) media, the predominant Ni-citrate complex constituted 45.58% and 74.92% and the Ni-lactate complex constituted 23.10% and 8.02%, respectively. These results indicated that the presence of citrate led to a great reduction of free Ni^2+^. Figure 1 shows the variation of OD_660_ values and final SRB concentration in the four different treatments. In the absence of Ni, after a lag period of 0.5 day, the OD_660_ of the Ni-free control media increased rapidly and reached a maximum value within 4 days, and the final SRB concentration increased from 3.71 × 10^7^ cells/mL (initial SRB concentration in each treatment) to 1.65 × 10^9^ cells/mL. In the presence of 1 mM Ni without citrate, both OD_660_ and the final SRB concentration maintained the initial level with little change during the experiments. In the Ni/citrate (0.5) and Ni/citrate (1.0) media, OD_660_ reached to 0.12 and 0.14 within 4 day, respectively, over 20-times higher than the initial level. Meanwhile, the final SRB concentration increased greatly to 1.38 × 10^9^ and 1.92 × 10^9^ cells/mL, respectively. The above results showed that the biomass of the mixed culture and SRB increases significantly after the addition of citrate, suggesting that citrate can effectively alleviate Ni toxicity for all anaerobic bacteria, including SRB.

**Table 1:** Predicted nickel speciation in Ni/citrate (0), Ni/citrate (0.5) and Ni/citrate (1.0) media using the MINTEQ 3.0 model.

Media	% of Total Ni (1 mM)							
	Ni^2+^	NiCl^+^	NiSO_4__(aq)_	[Ni-Lactate]^+^	[Ni-(Lactate)_3_]^−^	Ni-(Lactate)_2__(aq)_	[Ni-Citrate]^−^	[Ni-(Citrate)_2_]^4−^
Ni/citrate (0)	26.20	0.03	16.18	43.21	0.714	13.67	–	–
Ni/citrate (0.5)	13.88	0.01	8.53	23.10	0.39	7.38	45.58	0.84
Ni/citrate (1.0)	4.79	–	2.92	8.02	0.14	2.58	74.92	6.63

**Figure 2:**
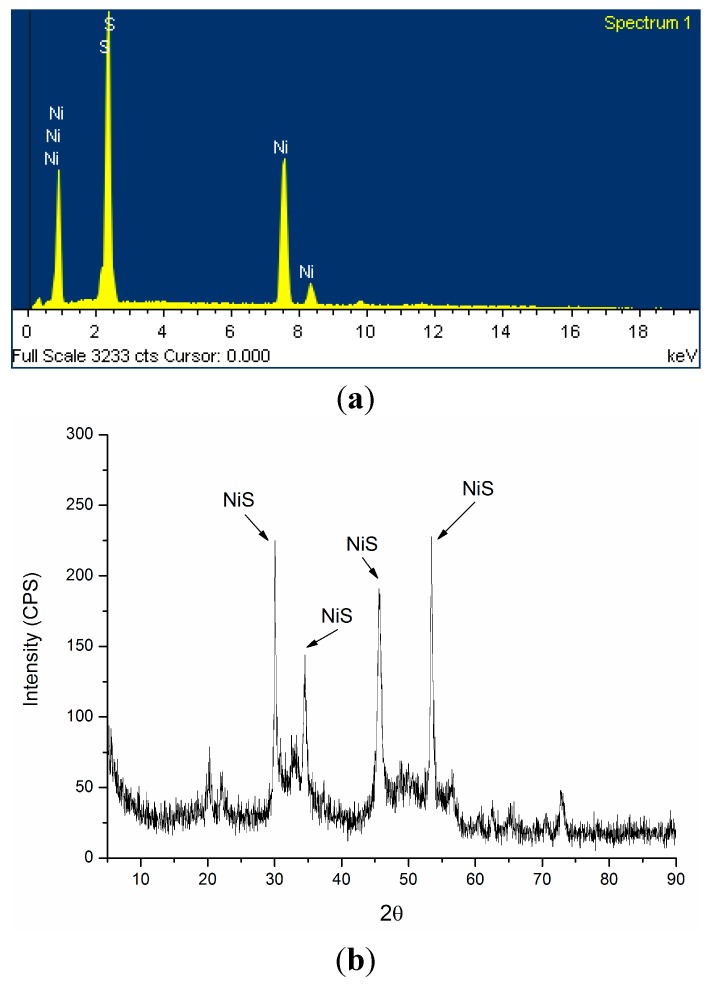
Energy dispersive X-ray spectroscopy (EDS) (**a**) and X-ray diffraction (XRD) (**b**) analysis of the precipitate.

Table 2 shows the final concentrations of the sulfate, dissolved sulfide, Ni, as well as final pH values in the four treatments. At the end of incubation, in the Ni-free control, Ni/citrate (0.5) and Ni/citrate (1.0) media, sulfate concentration decreased from 30.00 to 25.08, 24.26 and 24.00 mM, respectively. The pH values increased to 7.09, 7.23 and 7.18, respectively, except the Ni/citrate (0) media, the pH value of which remained at 6.77. No variation of sulfate and Ni was observed in Ni/citrate (0) media during the whole period of the experiment. At the end of the incubation, the black precipitate appeared at the bottom of the reactors containing the Ni/citrate (0.5) and Ni/citrate (1.0) media, and it was completely removed from the solutions. Energy dispersive X-ray spectroscopy (EDS) results demonstrated that the black precipitate was mostly composed of sulfur and nickel with a metal/sulfur ratio of 1.0 (Figure 2a). X-ray diffraction (XRD) analysis further confirmed that the metal crystals were NiS (Figure 2b).

**Table 2:** Final concentrations of sulfate, sulfide and Ni, as well as final pH values in the Ni-free control, Ni/citrate (0), Ni/citrate (0.5) and Ni/citrate (1.0) media.

Media	pH	Sulfate (mM)	Sulfide (mM)	Ni (mM)
Ni-free control	7.09 ± 0.31	25.08 ± 1.57	1.44 ± 0.04	–
Ni/citrate (0)	6.77 ± 0.23	29.96 ± 1.46	0.08 ± 0.01	0.98 ± 0.04
Ni/citrate (0.5)	7.23 ± 0.18	24.26 ± 1.26	1.50 ± 0.03	0
Ni/citrate (1.0)	7.18 ± 0.15	24.00 ± 1.31	1.63 ± 0.05	0

### 2.2. Mechanism of Ni^2+^ Masking and Removal

**Figure 3:**
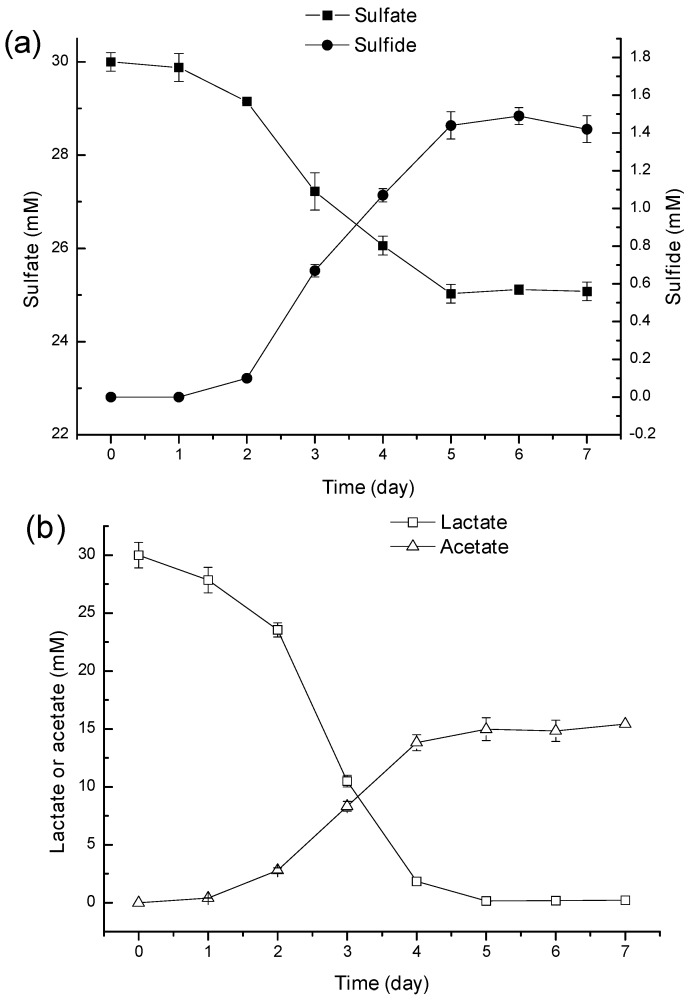
Residual concentrations of sulfate and sulfide (**a**) and lactate and acetate (**b**) in Ni-free lactate media with time (error bars represent one standard deviation of the mean; *n* = 3).

Figure 3 shows the variations of sulfate, sulfide, lactate and acetate concentration in the citrate/Ni-free lactate medium. The sulfate concentration decreased to 25.08 mM at the rate of 0.98 mmol/L·day within five days, while the dissolved sulfide concentration increased to 1.44 mM (Figure 3a). At the same time, lactate was completely consumed, and the acetate concentration increased to 15 mM (Figure 3b). Acetate was the main metabolic product from lactate in the Ni/citrate-free lactate medium. The simultaneous decline of sulfate and lactate suggested that lactate served as the electron donor for sulfate reduction (Equation (3)) [15].

(3) $$2\text{ Lactate}+{\text{SO}}_{4}^{2-}\;\to 2\text{ Acetate}+{\text{HS}}^{-}+2{\text{ H}}^{+}+2{\text{ HCO}}_{3}^{-}$$

**Figure 4:**
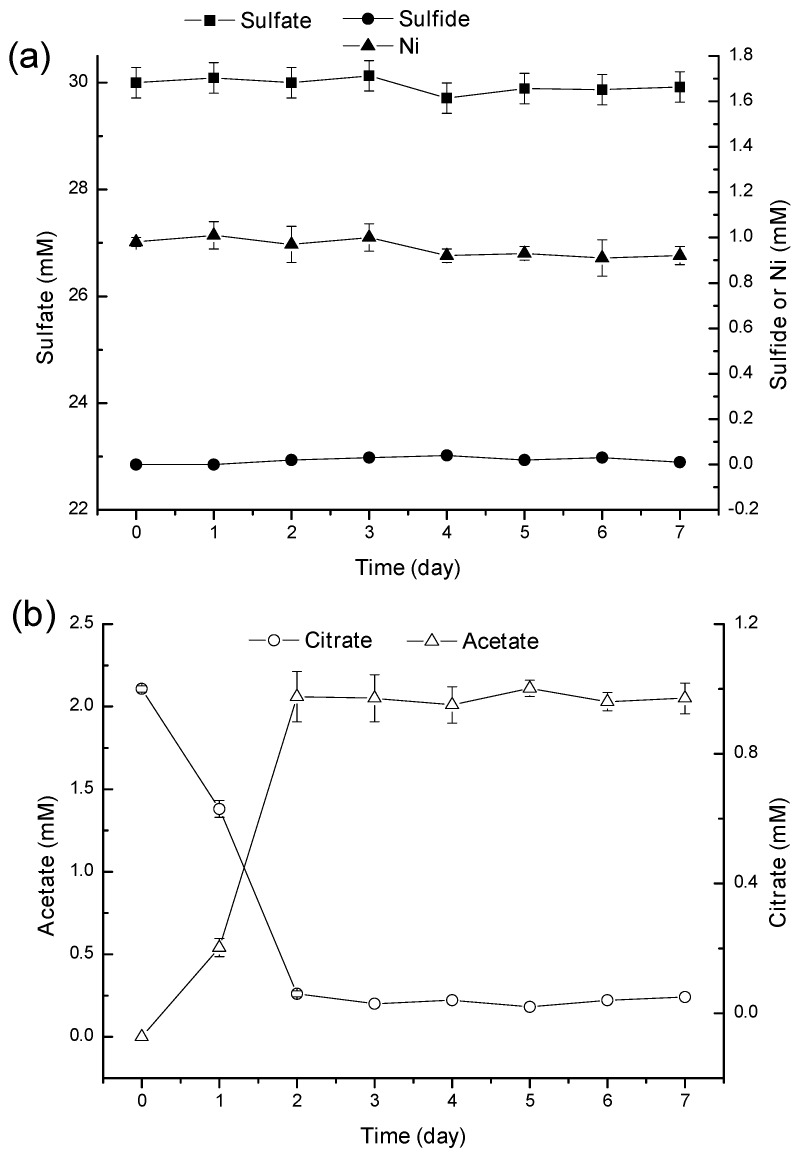
Residual concentrations of sulfate, sulfide and Ni (**a**) and citrate and acetate (**b**) in lactate-free Ni/citrate media with time (error bars represent one standard deviation of the mean; *n* = 3).

Figure 4 showed that in the lactate-free Ni/citrate medium, all sulfate still remained in the media during the experiments, and dissolved sulfide was not detected, indicating that sulfate reduction does not occur without lactate as the electron donor. Therefore, Ni was not removed from the solution (Figure 4a). Whereas citrate was completely consumed within 2 days, the acetate concentration increased to 2.25 mM and remained relatively constant till the end of the experiments (Figure 4b), demonstrating that citrate and acetate do not serve as electron donors of sulfate reduction in this bioprocess.

**Figure 5:**
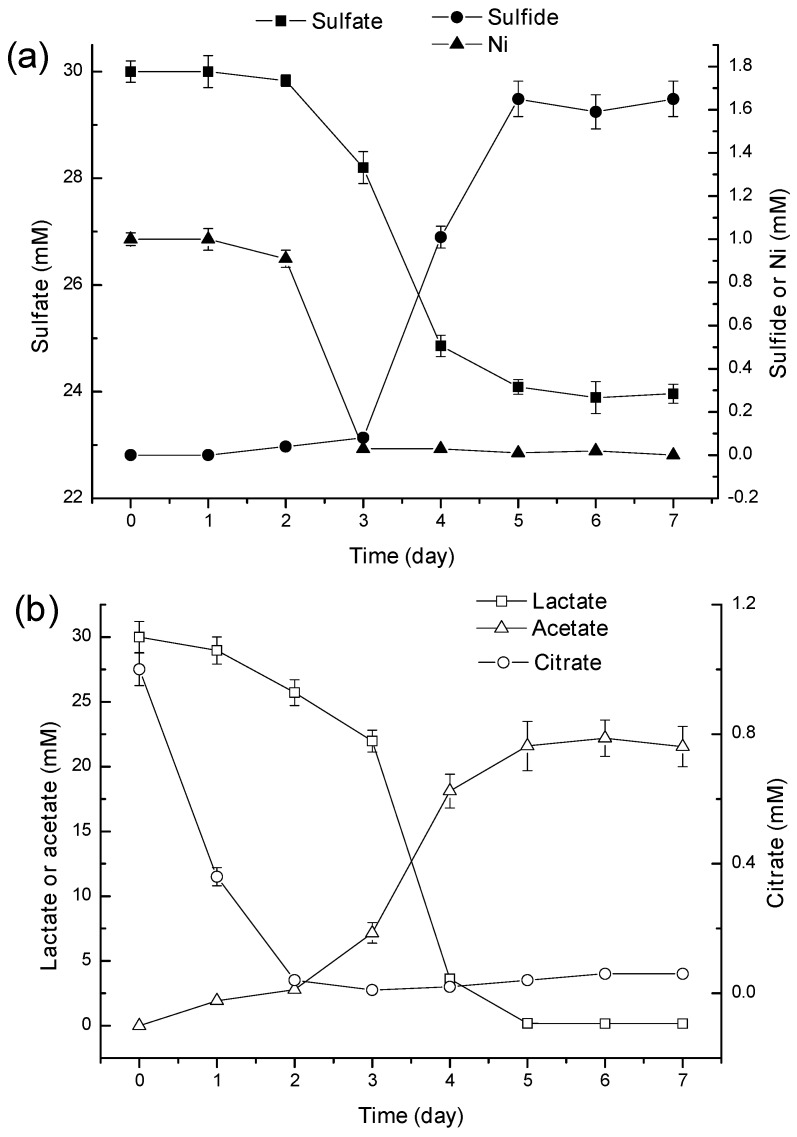
Residual concentrations of sulfate, sulfide and Ni (**a**) and lactate, citrate and acetate (**b**) in the lactate-Ni/citrate media with time (error bars represent one standard deviation of the mean; *n* = 3).

Figure 5 shows that in the lactate-citrate/Ni medium, the sulfate concentration decreased to 24.09 mM within 5 days at the rate of 1.2 mmol/L·day (Figure 5a). Meanwhile, Ni was completely removed from solutions within 3 days, and a black precipitate was observed at the bottom of the reactor. After the complete removal of Ni, the dissolved sulfide level increased to 1.65 mM within the following two days and remained constant to the end of the experiments (Figure 5a). Without lag periods, lactate and citrate were completely consumed within five and two days at the rate of 6.0 and 0.5 mmol/L·day, respectively (Figure 5b). Acetate concentration increased to 21.59 mM at the end of the incubation (Figure 5b).

### 2.3. Discussion

The bioavailability of heavy metal is critically dependent on its speciation, and it is generally assumed that free metal ions are most toxic to microorganisms [16,17]. It was reported that Ni was not toxic to *P. fluorescens* and *P. alcaliphila* when it was complexed with citrate [12,13]. According to the data obtained from MINTEQ3.0, Ni-citrate and Ni-lactate complexes, free Ni^2+^, NiSO_4__(aq)_ and NiCl^+^ were present in the Ni/citrate (zero), Ni/citrate (0.5) and Ni/citrate (1.0) media, but it is unknown whether Ni-lactate complexes NiSO_4__(aq)_ and NiCl^+^ had adverse effects for SRB growth. Compared with that in the Ni/citrate (0) media, the concentration of free Ni^2+^ in the Ni/citrate (0.5) and Ni/citrate (1.0) media decreased greatly due to the formation of Ni-citrate complexes. The toxic concentration of Ni^2+^ for SRB consortium in the batch test was 20 mg/L [18], but Poulson *et al.* found that a Ni^2+^ concentration greater than 10 mg/L exerted a marked toxic effect to *Desulfovibrio desulfuricans* [19]. It was noted in this study that free Ni^2+^ concentration in the Ni/citrate (0.5) and Ni/citrate (1.0) media was lower than the previously reported toxic concentration (20 mg/L). This might be due to the formation of a stable Ni-citrate complex, resulting in the decline of the free Ni^2+^ in solutions, alleviating the inhibition of Ni^2+^ on SRB growth. This result was consistent with that of Jong *et al.* [20] and Kieu *et al.* [8], who investigated the removal of heavy metals by SRB in semi-continuous stirred tank and bench-scale upflow anaerobic packed bed reactors and found that heavy metal toxicity for SRB might be alleviated via citrate complexation. It should also be noted that the initial free Ni^2+^ concentration in Ni/citrate (0.5) medium (about 0.14 mM) was relatively higher than that in Ni/citrate (1.0) medium (0.05 mM); whereas there was no significant difference in bacterial growth and the final SRB concentration between the two treatments. This may be attributed to the resistance of SRB itself to 0.14 mM Ni^2+^ in the Ni/citrate (0.5) media.

In our study, the precipitate was composed of NiS only; Metal hydroxides or carbonates crystalline phases were not found. The possibility of Ni removal by hydroxide precipitation from a pH increase and carbonate precipitation from an alkalinity increase can be eliminated. In the Ni/citrate (0) media, bacterial growth and metabolism were completely inhibited, and the solution pH remained unchanged. In the Ni-free control, Ni/citrate (0.5) and Ni/citrate (1.0) media, the pH increase may be caused by the alkalinity increase from microbial metabolism [21]. Moreover, it can also be noted that the sulfate reduction efficiencies in the Ni/citrate (0.5) (19%) and Ni/citrate (1.0) (20%) media were both relatively higher than that in Ni-free control media (16.4%). This may be attributed to the bacterial citrate metabolism from the Ni-citrate complex.

In the range from pH 5 to pH 8, Ni and citrate are generally present as a mononuclear bidentate [NiCit]^−^ complex, which could be biodegraded by bacteria under aerobic conditions [12,13]. According to MINTEQ3.0, 99.5% of the 1 mM Ni was present in the form of the bidentate Ni-citrate complex in the lactate-free Ni/citrate medium. Citrate consumption suggested that the Ni-citrate complex can be biodegraded under anaerobic conditions, and acetate was the sole metabolic product from the Ni-citrate complex (Equation (4)) [9]. In our study, although bacteria could grow utilizing citrate as a carbon source, acetate could not serve as the electron donor for sulfate reduction, which was different from that reported by Gamez and Stams [9,10].

(4) $${\left[\text{Ni}-\text{citrate}\right]}^{-}\to {\text{Ni}}^{2+}\;+2\text{ Acetate}+{\text{H}}_{2}+2{\text{ CO}}_{2}$$

In the lactate-citrate/Ni media, acetate was the sole metabolism product from lactate and citrate, and Ni was completely removed from the solutions. The simultaneous decrease of sulfate and Ni concentrations suggested that Ni was mainly removed via precipitation with the produced sulfide. It was noteworthy that not until complete removal of Ni did the accumulation of sulfide commenced, and the accumulation of sulfide ceased after the complete consumption of lactate. This suggested that oxidation of lactate to acetate serving as the electron donor may be responsible for the sulfate reduction.

Acetate is a widely-used electron donor for sulfate reduction [2,22]. However, this work suggested that acetate did not serve as the electron donor for sulfate reduction in our treatments, which could be due to the inhibition of acetate-consuming bacteria, either by sulfide or by acetate [23], or by Ni^2+^ ions in the reactors; whereas the lactate-utilizing SRB was not inhibited.

## 3. Materials and Methods

### 3.1. Sulfate-Reducing Bacteria (SRB) Enrichment

A mixed culture of SRB using lactate as a carbon source was enriched from the anaerobic sludge collected from a wastewater treatment plant in Chengdu, China. The enrichment medium containing 1.0 g/L of sulfate ions had the following composition in 1 L of deionized water: 1.12 g glycerol 2-phosphate, 1 g NH_4_Cl, 0.85 g MgSO_4_·7H_2_O, 1 g Na_2_SO_4_, 0.1 g yeast, 3.2 g bromo-ethane-sulfonic-acid (BESA) and 3.2 g sodium lactate. Magnesium sulfate and sodium sulfate were used as sulfate sources, and glycerol 2-phosphate was used to provide phosphate for cell synthesis. BESA was added to the culture to inhibit methanogenic activity [24]. In brief, 5 g of anaerobic sludge were inoculated into 100 mL of the enrichment medium and incubated anaerobically on a rotary shaker (120 rpm) at 30 °C. The sulfate reduction was measured via the analysis of sulfate and sulfide. When the dissolved sulfide concentration increased to 50 mg/L, 10 mL of the culture were transferred into fresh enrichment medium for further enrichment. After 8 continuous transfers, the mixed culture of SRB was obtained by centrifugation (2500× *g*, 10 min) and washed 2–3 times with a 20-fold dilution of the enrichment medium. The SRB culture was kept at 4 °C as the inoculum for further studies.

### 3.2. Batch Experiments

Batch experiments were carried out in serum bottles (500 mL) containing 450 mL of the defined mineral medium. The bottles were sparged with N_2_ for 15 min to provide a reducing atmosphere. All bottles were sealed with rubber stoppers and aluminum crimps after the addition of 50 mL of SRB inoculum having 0.5 OD_660_ and were incubated at 30 °C under shaking at 100 rpm.

### 3.3. Defined Mineral Media

All media contained the sulfate medium (g/L): Glycerol 2-phosphate, 0.3; NH_4_Cl, 0.3; Na_2_SO_4_, 4.26; yeast, 0.05; MgCl_2_, 0.05; BESA, 3.2. NiCl_2_·6H_2_O was used as the source of Ni, and lactate and citrate were added in the form of sodium lactate and sodium citrate. The addition amounts of Ni, lactate and citrate in defined mineral media were calculated according to the experiment demands. Solution pH was adjusted to 6.8 with HCl or NaOH.

### 3.4. Effect of Citrate on the SRB Growth and Ni^2+^ Removal

For the toxicity masking experiments, the four treatments were as follows: (1) Ni-free control: 30 mM lactate; (2) Ni/citrate (0): 30 mM lactate + 1.0 mM Ni^2+^; (3) Ni/citrate (0.5): 30 mM lactate + 0.5 mM citrate + 1 mM Ni^2+^; (4) Ni/citrate (1.0): 30 mM lactate + 1.0 mM citrate + 1.0 mM Ni^2+^. Speciation of Ni in Ni/citrate (0), Ni/citrate (0.5) and Ni/citrate (1.0) was verified using the MINTEQ3.0 program. Glycerol 2-phosphate and bromo-ethane-sulfonic-acid were not found in the database and were not included. Thermodynamic constants in the MINTEQ3.0 database were used in the calculations. Samples were withdrawn periodically for analysis of OD_660_ to monitor bacterial growth. At the end of the experiment, samples were taken for analysis of nickel, sulfate, sulfide, as well as quantification analysis of SRB. Each treatment had three replicates.

### 3.5. Biodegradation of Ni-Citrate Complex

Three treatments were conducted as follows: (1) Citrate/Ni-free lactate medium: 30 mM lactate; (2) Lactate-free citrate/Ni medium: 1.0 mM citrate + 1.0 mM Ni^2+^; (3) Lactate-citrate/Ni medium: 30 mM lactate + 1.0 mM citrate + 1.0 mM Ni^2+^. Samples were withdrawn periodically for the analysis of sulfate, sulfide, Ni and organic acids. Each treatment had three replicates.

### 3.6. Analytical Methods

The microbial population density was determined spectrophotometrically at 660 nm (TU-1810 UV-Vis Spectrophotometer, Purkinje General Instrument Co., Beijing, China). Sulfate was measured by ion chromatography (Metrohm 761 Compact IC, Metrohm Ltd., Herisau, Switzerland). Sample separation and elution were performed using a Metrosep A Supp 5–150 analytical column (Metrohm Ltd., Herisau, Switzerland) with carbonate/bicarbonate eluent (3.2 mM Na_2_CO_3_/1.0 mM NaHCO_3_ at 0.7 mL/min). Quantitative determination of sulfide was performed calorimetrically by the methylene blue method [25]. Ni was determined by atomic absorption spectrometry (AAS, Z-2300, Hitachi, Japan). Lactate, acetate and citrate were analyzed by HPLC equipped with a Hi-Plex H column and an RID detector set at 45 °C (Agilent Technologies, Palo Alto, CA, USA). The metal sulfide precipitate was characterized by energy dispersive X-ray spectroscopy (EDS, IE 150, Oxford Instrument, Oxfordshire, UK) and X-ray diffraction (XRD, DX2500, Fangyuan Instrument Co., Ltd., Dandong, China) analysis.

Quantification of SRB was conducted by quantitative real-time PCR (q-PCR). Samples (about 0.02 g) from each treatments were washed three times with phosphate-buffered saline and centrifuging each preparation at 14,000× *g*; Then, DNA was extracted using genomic DNA using the Soil DNA isolation Kit (MO BIO Laboratories, Carlsbad, CA, USA) according to the manufacturer’s instructions. The SRBs were quantified by an evaGreen assay targeting the dsrAB genes using a primer set: DSR1-F+ (5'-ACS CAC TGG AAG CAC GGC GG-3') and DSR-R (5'-GTG GMR CCG TGC AKR TTG G-3') (the expected product size is 221 bp) [26]. The q-PCR amplification was performed in a C1000 touch thermal cycle equipped with a CFX 384 Real-Time system (Biorad, Hercules, CA, USA). Results were expressed as the number of cells per milliliter of culture, assuming that the SRB contained only one copy of *dsrAB* gene per cell [27].

## 4. Conclusions

These data provide the first evidence of promoting Ni removal by masking Ni^2+^ toxicity to SRB via citrate complexation in batch reactors. In the presence of citrate, Ni-citrate complex formation led to the decline of free Ni^2+^ in solution, lessening Ni toxicity for and inhibition of SRB. In the biodegradation process, the Ni-citrate complex was biodegraded to Ni^2+^ and acetate, and sulfate was reduced to sulfide by SRB using lactate as the electron donor. The NiS precipitate was then formed, and Ni^2+^ was completely removed from the solution. Therefore, when wastewater contains a relatively high concentration of Ni^2+^, it is feasible to promote Ni removal by masking Ni^2+^ toxicity to SRB via citrate complexation in the single-stage SRB-based bioprocess. This approach offers a potential alternative to increase the removal of other heavy metals from wastewater, such as Cu^2+^, Zn^2+^, Pb^2+^, *etc.*
